# Association Between Liver Function Grade and Post‐Hepatectomy Liver Failure in Patients With Hepatocellular Carcinoma: A Latent Class Analysis

**DOI:** 10.1002/ags3.70138

**Published:** 2025-11-25

**Authors:** Ling Liu, Jintao Zheng, Ye Wang, Chenao Yang, Jiachen Zhang, Changku Jia

**Affiliations:** ^1^ Department of Hepatobiliary and Pancreatic Surgery Hangzhou First People's Hospital Affiliated to Medical School of Westlake University Hangzhou Zhejiang China; ^2^ Department of Hepatobiliary and Pancreatic Surgery The Fourth School of Clinical Medicine, Zhejiang Chinese Medical University Hangzhou Zhejiang China; ^3^ Department of Gastroenterological Surgery Hangzhou First People's Hospital Affiliated to Medical School of Westlake University Hangzhou Zhejiang China; ^4^ Department of Hepatobiliary and Pancreatic Surgery The Second Affiliated Hospital, School of Medicine, Zhejiang University Hangzhou Zhejiang China

**Keywords:** hepatocellular carcinoma, latent class analysis, liver function, post‐hepatectomy liver failure

## Abstract

**Objective:**

Hepatocellular carcinoma (HCC) is a leading cause of cancer mortality, with post‐hepatectomy liver failure (PHLF) representing a major complication. We aimed to develop an objective, multidimensional liver function grading system via latent class analysis (LCA) and assess its predictive value for PHLF in combination with surgical parameters.

**Methods:**

Among 185 HCC patients who underwent resection, the LCA incorporated nine liver function indicators, including severity of liver cirrhosis, splenomegaly, platelet count (PLT), etc., to categorize liver function into three grades: poor, intermediate, and good. Discrimination was evaluated using entropy. Restricted cubic spline analysis assessed the association between blood loss and PHLF, and multivariate logistic regression identified independent predictors.

**Results:**

LCA yielded three distinct classes (entropy = 0.953). Blood loss showed a nonlinear relationship with PHLF (*p* = 0.012), with a risk threshold at 150 mL. PHLF incidence was 36.6% in poor versus 11.5% in good liver function. Liver function grade and resection extent independently predicted PHLF, with consistent effects across resection subgroups (interaction *p* > 0.05).

**Conclusion:**

This LCA‐based grading system, integrating imaging and biochemical markers, enables precise PHLF risk stratification and, combined with surgical variables, may inform individualized perioperative management in HCC resection.

## Graphical abstract

We retrospectively analyzed clinical data from patients who underwent hepatectomy for hepatocellular carcinoma (HCC) using LCA‐based grading system. These findings provide a new risk stratification framework for the design of precision surgery to treat patients with HCC.

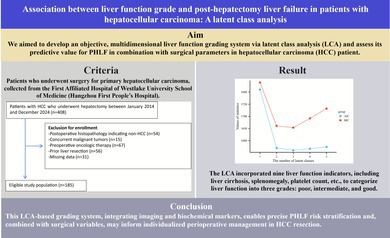

## 1 Introduction

Hepatocellular carcinoma (HCC) is a leading cause of global cancer‐related deaths. Partial hepatectomy has long been the only curative treatment of HCC [1, 2]. However, post‐hepatectomy liver failure (PHLF) is a common and serious complication that worsens patient outcomes. Its high incidence (0.7%–39.6%) and fatality (approximately 50%) rates contribute to its role as the most common cause of perioperative mortality among HCC patients undergoing major hepatectomy [3, 4, 5]. Therefore, a thorough assessment of preoperative liver function is crucial. Traditional evaluation systems, such as the Child‐Pugh classification and Model for End‐Stage Liver Disease (MELD) score, while widely used in clinical practice, can only reflect baseline liver function but struggle to accurately predict the risk of PHLF [6, 7]. They also fail to quantify the impact of liver fibrosis on surgical tolerability. This evaluation gap often leads to misclassification of patients with borderline liver function, thus impeding surgical decision‐making.

Non‐invasive liver fibrosis detection technologies, such as 2D shear wave elastography (2D‐SWE), have introduced new dimensions to liver function assessment [8]. Liver stiffness (LS) values are associated with the risk of PHLF, but their predictive power is limited by the use of a single indicator. Indocyanine green (ICG) clearance tests are widely used dynamic liver function assays. A higher ICG retention at 15 min (ICG R15) value or a lower ICG plasma disappearance rate value indicates a higher risk of PHLF, which is related to the extent of hepatectomy. It is generally believed that patients with normal liver function (ICG R15 ≤ 10%) can tolerate two or more segmentectomies. However, the credibility of ICG clearance tests may be reduced by abnormal hepatic perfusion, intrahepatic shunting, hyperbilirubinemia, and biliary obstruction [9, 10]. Meanwhile, LCA, an emerging machine learning method, can differentiate subgroups with similar characteristics by integrating multidimensional data, and shows unique advantages in stratifying patients with chronic liver disease [11, 12]. However, to our knowledge, LCA has not been applied to systematically explore the mechanisms linking liver function classification and PHLF; furthermore, coordinated analyses of imaging features, laboratory indicators, and clinical parameters are lacking. The innovation of this study lies in its identification of key gaps in the current PHLF prediction system: first, traditional evaluation methods fail to reflect the heterogeneity of liver function status, thus hindering risk stratification; second, the collaborative predictive value between LS measurement and routine biochemical and Hepatitis B Virus (HBV) indicators has not been clearly defined, necessitating the establishment of a predictive model based on multimodal data. To address these issues, this study adopted a research strategy combining a retrospective cohort design with advanced statistical methods. By integrating seven liver function status indicators to construct an LCA model, we overcame the subjective limitations of traditional classification and used restricted cubic splines analysis to reveal a nonlinear relationship between intraoperative blood loss and PHLF. Furthermore, the independent contribution of each risk factor was determined by multiple regression and interaction tests.

This study aimed to establish a precision liver function stratification system based on LCA to further elucidate the interaction between the extent of hepatectomy and liver function status. Ultimately, we sought to construct a multi‐dimensional prediction model for PHLF, providing an objective basis for the individualized design of surgical therapy for HCC patients. By integrating imaging and clinical data, the study also aimed to build a foundation for liver function assessment in the digital medicine era.

## 2 Materials and Methods

### 2.1 Study Population

**FIGURE 1:**
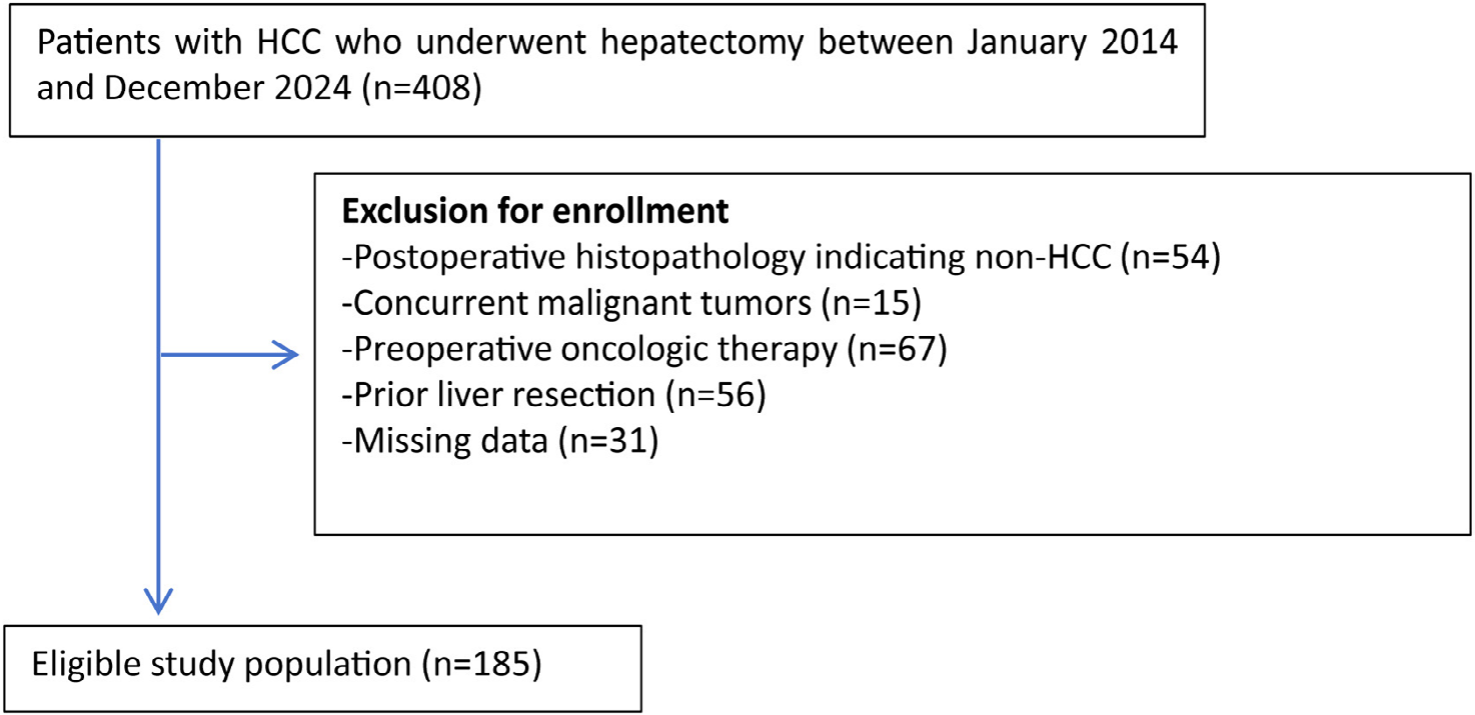
Selection of the study population.

We conducted a retrospective analysis of 185 HCC patients who underwent radical surgery at the First Peoples Hospital of Hangzhou, affiliated with the Medical College of West Lake University, from January 2014 to December 2024. Inclusion criteria were: (1) Postoperative histopathologic confirmation of HCC; (2) No extrahepatic or distant metastasis; (3) No concurrent malignant tumors in other organs; (4) No prior oncologic therapy; (5) Complete clinical data. Exclusion criteria were: (1) Postoperative pathology indicating non‐HCC; (2) Concurrent malignant tumors; (3) Preoperative oncologic therapy; (4) History of prior liver resection; (5) Missing data. A flow diagram illustrating patient selection is shown in Figure 1.

### 2.2 Data Collection

We collected the following patient data: demographic information (age, sex, body mass index [BMI]); perioperative laboratory test results including serum total bilirubin (TB), albumin (ALB), and alpha‐fetoprotein (AFP) levels; prothrombin time (PT); PLT; HBV status; HBV Deoxyribonucleic Acid (DNA) level; and ICG R15 result; tumor‐related data such as tumor size and number; surgical data including intraoperative blood loss and extent of liver resection; hepatic contrast enhanced computed tomography (CT) results including the extent of liver cirrhosis and splenic dimensions; and ultrasound imaging findings, including LS and splenic dimensions.

### 2.3 LS Assessment by Two‐Dimensional Shear Wave Elastography

All patients underwent a 2D‐SWE liver examination using the Aixplorer ultrasound imaging system (Supersonic Imagine, Aix‐en‐Provence, France) equipped with an SC6‐1 convex array probe. Scans were performed by one of three experienced ultrasound technicians. According to the guidelines of the European Federation of Ultrasound in Medicine and Biology, patients were required to fast for at least 4 h before the examination [13]. During the examination, patients were placed in a supine position with their right arm maximally abducted. The intercostal approach was used to assess the right hepatic lobe. Patients were instructed to hold their breath for at least 5 s to obtain stable SWE images. Technicians placed at least five circular regions of interest (ROIs), each approximately 15.0 mm in diameter, in the color box of the elastography image perpendicular to the liver surface, with a depth of at least 2.0 cm. The mean value of each ROI measurement and the median of multiple ROI measurements were used to determine the patient's overall Liver Stiffness Measurement (LSM) 2D‐SWE value. Based on LSM 2D‐SWE, fibrosis stages were defined as: F0: < 6.430 kPa; F1: 6.430 ~ 7.249 kPa; F2: 7.250 ~ 8.408 kPa; F3: 8.409 ~ 10.046; F4: ≥ 10.047 kPa.

### 2.4 Definitions

Splenomegaly was defined as a splenic length greater than 12 cm and a thickness greater than 4 cm on ultrasound examination or CT scan. Liver cirrhosis was diagnosed by two experienced radiologists on the basis of hepatic and extrahepatic secondary findings on CT scan. Hepatectomies were categorized as either small‐scale (involving no more than 2 Couinaud segments) or large‐scale (involving 3 or more Couinaud segments) liver resections. Surgical specimens were submitted for histopathological examination to diagnose liver cirrhosis and HCC. PHLF was defined as a TB level exceeding 50 μmol/L on or after postoperative day 5 and a PT index < 50% (equivalent to an international normalized ratio [INR] > 1.7). The International Study Group of Liver Surgery (ISGLS) further classifies the severity of PHLF into three grades, with grade A indicating a temporary, minor deterioration in liver function that does not require invasive treatment; Grade B indicates a deviation from the expected result but still manageable and not requiring invasive treatment; Grade C indicates the presence of severe liver and multiple organ failures requiring invasive treatment [14]. To reflect clinical severity, we dichotomized PHLF as follows: PHLF grade 1 = ISGLS Grades B‐C; PHLF grade 0 = no PHLF or ISGLS Grade A (transient, non‐invasive).

### 2.5 Liver Function Status by LCA Group

We used LCA to identify new subgroups. LCA is a finite mixture modeling method that fits a series of models to data, assuming that the observed multivariate distribution is a mixture of several distributions. The advantage of LCA is its ability to define subgroups by considering multiple variables simultaneously without focusing on outcomes. Prior to LCA, we excluded observations with missing values. Models were estimated via maximum likelihood using an EM algorithm, with convergence criteria set to a maximum of maxiter = 1000 iterations and a tolerance of tol = 1e‐10. To mitigate local optima, each candidate model employed nrep = 100 random starts, and the best‐fitting solution was retained. The number of classes was determined by systematically comparing 1–5 class solutions based on Akaike's Information Criterion (AIC), the Bayesian Information Criterion (BIC), entropy, and mean posterior probability (Mean PP), alongside interpretability and a minimum class‐size threshold (> 5%). After assigning participants to the most likely category by using LCA, we analyzed summary statistics (mean, median, and proportion) of baseline clinical and biological characteristics for each category to evaluate the distinguishing features of the identified categories. We included indicators related to liver function status in the LCA classification, such as cirrhosis, splenomegaly, PLT, ALB, TB, PT, ICG R15 results, HBV status and degree of liver fibrosis. Based on item response probability, we identified three potential categories, representing groups with poor, intermediate, and good liver function.

### 2.6 Statistical Analysis

Continuous variables with a normal distribution were represented by mean ± standard deviation (mean ± SD) and tested using the Student's t‐test; continuous variables with a non‐normal distribution were represented by median (interquartile range) and tested using the Mann–Whitney *U*‐test. Categorical variables were represented by frequency (%) and compared using Pearson *χ* ^2^ or Fisher's exact tests. We used restricted cubic splines at the 5th, 35th, 50th, 65th, and 95th percentiles to flexibly model the relationship between blood loss and PHLF. We then used a logistic proportional hazards regression model to estimate the risk ratio and 95% confidence interval for outcomes related to liver function status and the extent of liver resection. We further stratified the analysis by potential categories of liver function status to explore the relationship between the extent of liver resection and PHLF outcomes in different patient subgroups with varying liver function statuses. *p* < 0.05 indicated a statistically significant difference. All statistical analyses were performed using R software (v.4.3.3; http://www.r‐project.org/) and SPSS (version 26.0; IBM Corporation, Armonk, NY, USA).

## 3 Results

### 3.1 LCA Results With Liver Function

**FIGURE 2:**
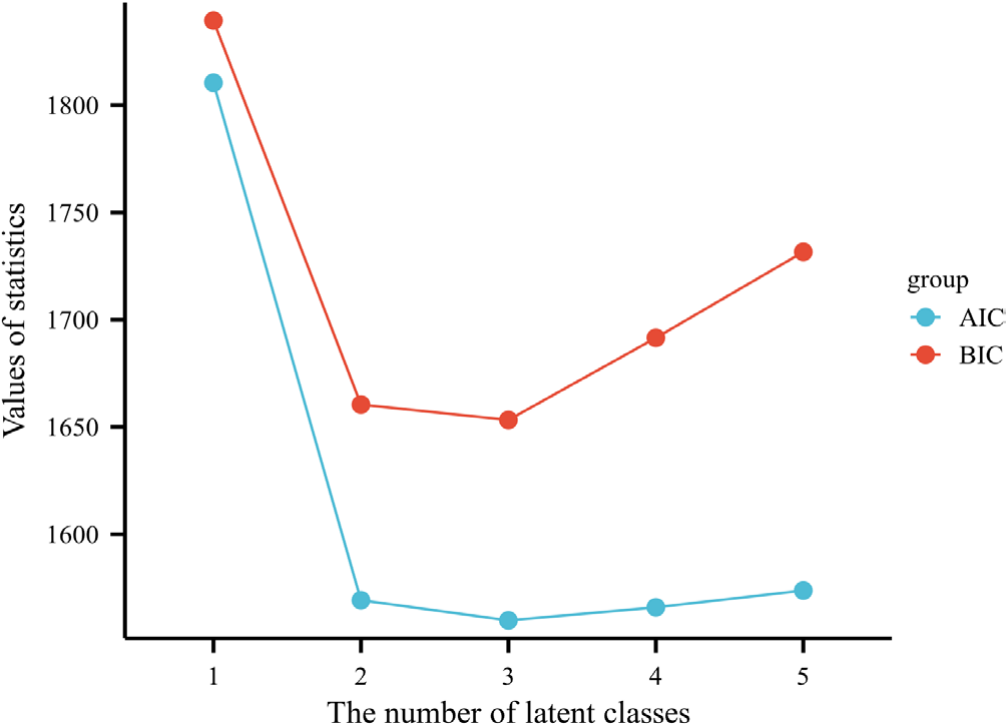
Akaike (AIC) and Bayesian (BIC) information criterion groups in models with different numbers of latent classes.

Baseline demographic and clinical characteristics of study participants are shown in Table 1. LCA showed that the three‐category model best fitted the data (Figure S1). As indicated by the fitting statistics, the Vuong‐Lo–Mendell‐ Rubin likelihood ratio test (VLMR) indicated that the three‐category model fit better than the two‐category and four‐category models, with an entropy value of 0.953. The Akaike information criterion value was 1559.87, and the BIC value was 1653.26, indicating good separation between categories (Figure 2, Table S1). The average potential category probabilities for Category 1, Category 2, and Category 3 were 0.962, 0.908, and 0.973, respectively. For simplicity, these three groups were referred to as poor, intermediate, and good liver function groups, comprising 41 (22.2%), 66 (35.6%), and 78 (42.2%) subjects, respectively. The poor liver function group was more likely to have a higher degree of liver cirrhosis; higher TB, ICG R15, and PT results; lower PLT and ALB values; splenomegaly; and HBV infection.

**TABLE 1:** Baseline characteristics of the study population.

Characteristics	Value
Sex (male: female)	148:37
Age (years)	57.5 ± 10.4 (32, 79)
BMI (kg/m^2^)	23.3 ± 3.1 (15.9, 32.9)
HBeAg positivity, *n* (%)	152, 82.2
HBV‐DNA level (IU/mL), *n* (%)	
< 200	141, 76.2
> 200	44, 23.8
Liver cirrhosis, *n* (%)	97, 52.4
Liver stiffness, *n* (%)	
F0‐1	39, 21.1
F2	34, 18.4
F3	27, 14.6
F4	85, 45.9
Splenomegaly, *n* (%)	69, 37.3
Platelet (10^3^/mm^3^)	151.1 ± 67.2 (45, 462)
PT (s)	12.4 ± 1.1 (10.5, 16.8)
Albumin (g/L)	41.1 ± 5.0 (24.5, 50.6)
Bilirubin (mg/dL)	17.1 ± 25.6 (5, 350)
AFP (μg/L)	1139.5 ± 4057.7 (1.1, 33696.2)
ICG‐R15 (%)	7.8 ± 4.5 (1.3, 21.6)
Tumor number, *n* (%)	
1	149, 80.5
2	26, 14.1
≥ 3	10, 5.4
Tumor size (largest) (cm)	3.8 ± 2.1 (0.7, 13)
Estimated blood loss (mL)	197.1 ± 160.0 (50, 800)
Range of resection (major hepatectomy), *n* (%)	33, 17.8
Surgical method (laparoscopy)	73, 39.5

### 3.2 Clinical Features

Baseline patient characteristics are summarized in Table 2 according to the LCA model based on the results of normality testing (Figure S2). PHLF was diagnosed in 15 (36.6%), 12 (18.2%), and 9 (11.5%) patients in the poor, intermediate, and good liver function groups, respectively. PHLF incidence was significantly higher in the poor liver function group than in the other two groups (*p* < 0.05). However, PHLF grades were similar between the three groups (*p* > 0.05). Baseline clinical and pathological data (including sex, age, BMI, AFP, and other laboratory indicators); tumor‐related data; and surgical data (number of tumors, maximum diameter of tumors, extent of resection, intraoperative blood loss) did not differ significantly between the three groups (*p* > 0.05).

**TABLE 2:** Baseline characteristics of participants according to liver function.

Class	1	2	3	*p*
*n*	41	66	78	
Sex (%)				
0	6 (14.6)	19 (28.8)	12 (15.4)	0.084
1	35 (85.4)	47 (71.2)	66 (84.6)	
Age (years), mean (SD)	58.76 (9.99)	55.80 (9.41)	58.37 (11.39)	0.238
BMI, mean (SD)	23.45 (2.79)	23.73 (3.23)	22.97 (3.19)	0.342
Tumor size (cm), median (IQR)	3.70 (2.20, 4.60)	3.00 (2.00, 4.47)	4.10 (2.82, 5.45)	0.055
*N* (%)				
1	31 (75.6)	51 (77.3)	67 (85.9)	0.499
2	7 (17.1)	12 (18.2)	7 (9.0)	
≥ 3	3 (7.3)	3 (4.5)	4 (5.1)	
AFP (μg/L), median (IQR)	8.80 (4.03, 55.67)	22.10 (3.11, 141.47)	44.86 (3.46, 492.97)	0.347
Surgical methods (%)				
0	25 (61.0)	38 (57.6)	49 (62.8)	0.812
1	16 (39.0)	28 (42.4)	29 (37.2)	
Range of resection (%)				
1	32 (78.0)	58 (87.9)	62 (79.5)	0.313
2	9 (22.0)	8 (12.1)	16 (20.5)	
Blood loss, mL, median (IQR)	150.00 (50.00, 200.00)	100.00 (100.00, 300.00)	200.00 (100.00, 300.00)	0.079
PHLF (%)	26 (63.4)	54 (81.8)	69 (88.5)	0.004
	15 (36.6)	12 (18.2)	9 (11.5)	
PHLF grade (%)	37 (90.2)	64 (97.0)	77 (98.7)	0.065
	4 (9.8)	2 (3.0)	1 (1.3)	

*Note:* Class: 1, 2, and 3 signify poor, intermediate, and good liver function groups, respectively. Sex: 0, female; 1, male. Surgical methods: 0, Open Surgery; 1, Laparoscopic surgery. Range of resection: 1, small‐scale; 2, large‐scale. Blood loss: 0, < 150 mL; 1, ≥ 150 mL. PHLF:0, no PHLF; 1, Presence of PHLF. PHLF Grade: 0, no PHLF or ISGLS Grade A; 1, ISGLS Grades B or C.

### 3.3 Risk Factor Prediction

**FIGURE 3:**
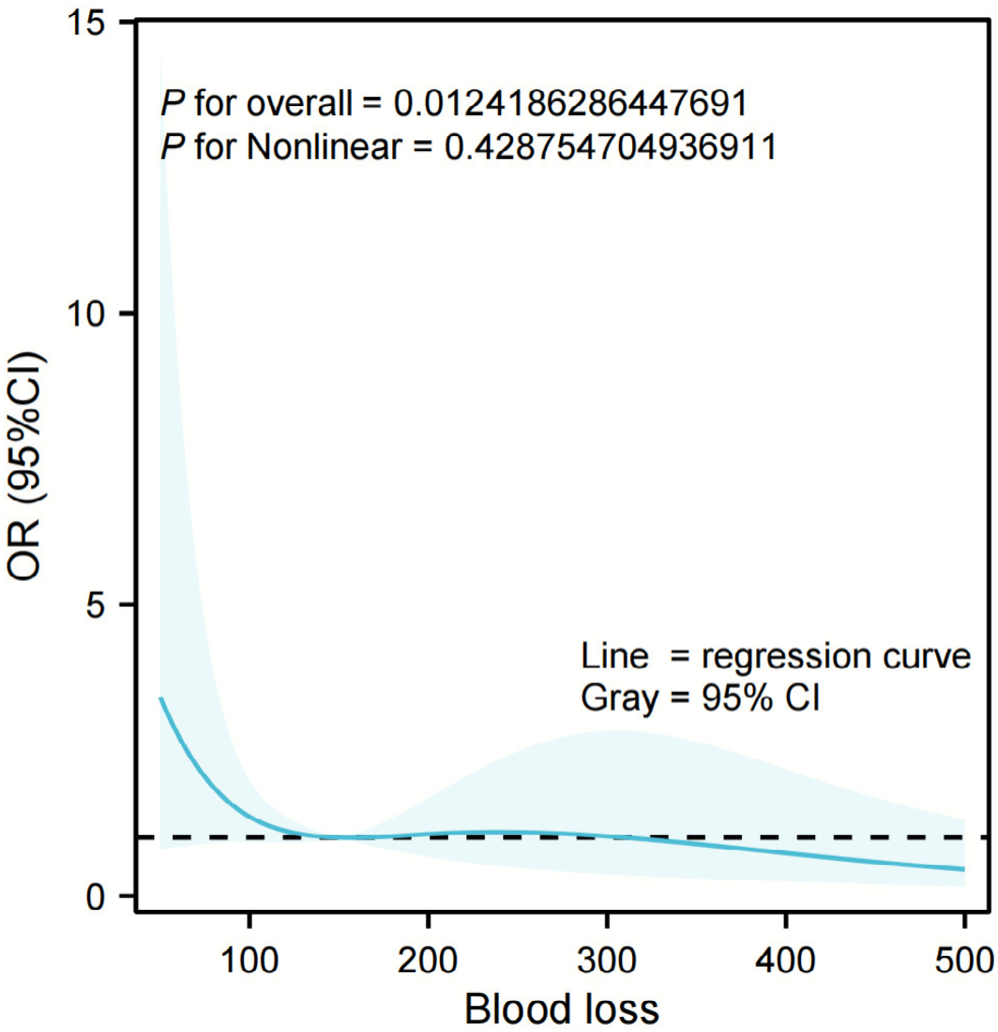
Restricted cubic spline modeling of the relationship between predicted blood loss and PHLF. Solid lines are fitted based on logistic regression. Shaded areas show 95% confidential intervals (CIs).

To identify the threshold value for intraoperative blood loss, we designed a restricted cubic spline model and visualized the relationship between predicted blood loss and PHLF. As shown in Figure 3, the odds ratio (OR) decreased before 150 mL of blood loss, then stabilized, and then increased rapidly (*p* overall = 0.012). Logistic regression identified statistically significant variables between the two groups, indicating that liver function grading and the range of resection are independent predictors of PHLF after liver resection in HCC patients (Table 3, all *p* < 0.05).

**TABLE 3:** Logistic regression analysis of risk factors of PHLF in HCC patients.

Characteristics	Total (*N*)	Univariate analysis		Multivariate analysis	
		Odds ratio (95% CI)	*p*	Odds ratio (95% CI)	*p*
Class	185				
1	41	Reference		Reference	
2	66	2.596 (1.064–6.333)	**0.036**	2.411 (0.837–6.942)	0.103
3	78	4.423 (1.725–11.339)	**0.002**	7.903 (2.336–26.733)	**< 0.001**
Sex	185				
1	148	Reference			
0	37	1.044 (0.417–2.614)	0.926		
Age	185	0.991 (0.956–1.026)	0.598		
BMI	185	0.978 (0.870–1.098)	0.702		
Tumor size	185	0.906 (0.785–1.047)	0.182		
Number	185				
1	149	Reference		Reference	
2	26	0.249 (0.102–0.610)	**0.002**	0.433 (0.139–1.348)	0.149
3	10	0.730 (0.146–3.660)	0.702	1.987 (0.298–13.234)	0.478
AFP	185	1.000 (1.000–1.000)	0.351		
Range of resection	185				
1	152	Reference		Reference	
2	33	0.112 (0.048–0.260)	**< 0.001**	0.125 (0.042–0.369)	**< 0.001**
Blood loss	185				
0	88	Reference		Reference	
1	97	1.976 (0.939–4.158)	0.073	1.866 (0.745–4.674)	0.183

*Note:* Class: 1, 2, and 3 signify poor, intermediate, and good liver function groups, respectively. Sex: 0, female; 1, male. Range of resection: 1, small‐scale; 2, large‐scale. Blood loss: 0, < 150 mL; 1, ≥ 150 mL. The bold values indicate statistical significance with *p* < 0.05.

### 3.4 Mediation Analysis of the Relationship Between Resection Range, Liver Function Grading, and PHLF Incidence

**FIGURE 4:**
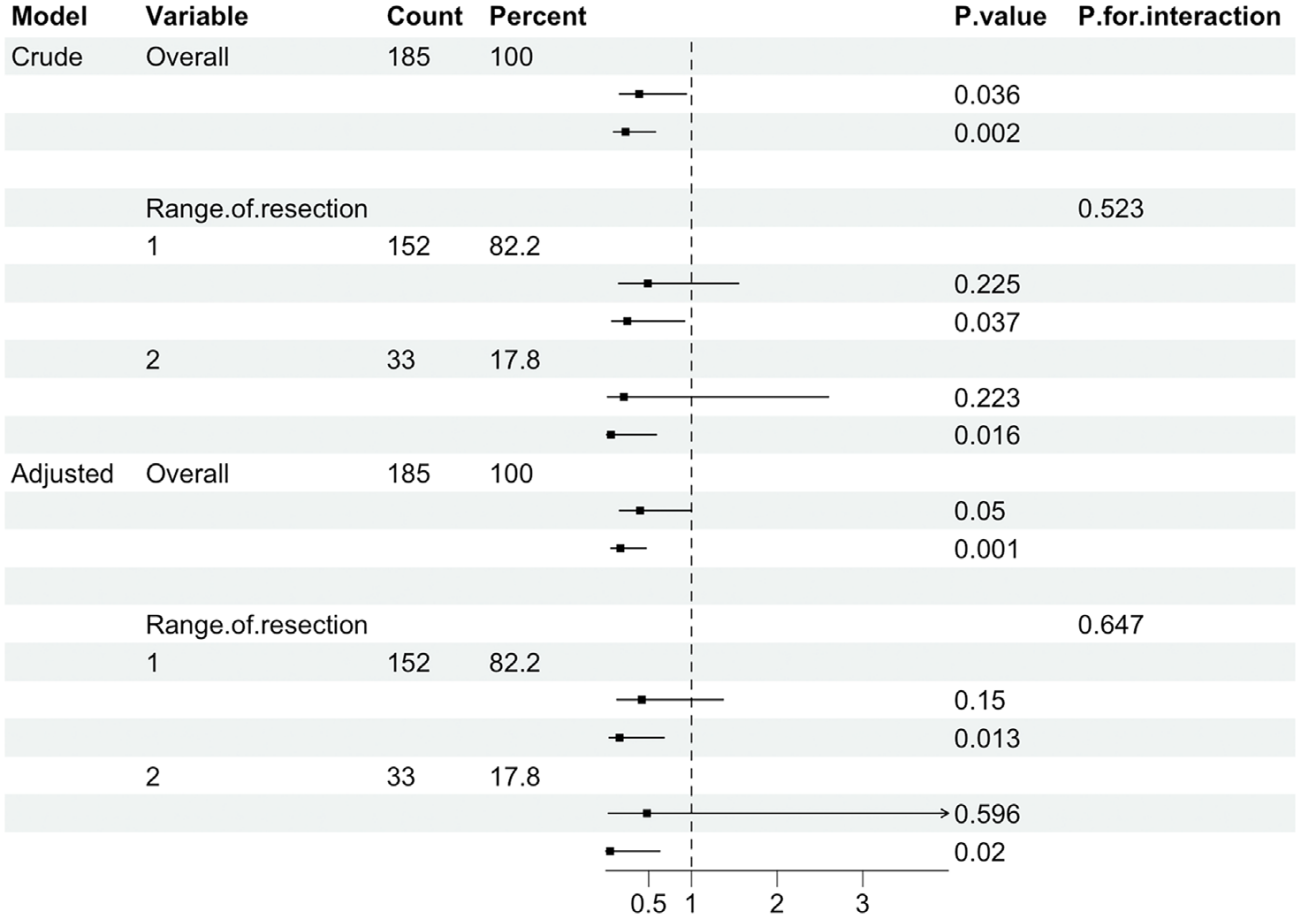
Association between liver function and risk of PHLF, stratified by range of resection. Crude and multivariable‐adjusted OR with 95% CI (adjusted sex, age, BMI, tumor size, blood loss).

We further stratified the analysis by potential categories of liver function to explore the relationship between liver function grading and PHLF in different resection range subgroups (Figure 4). Without adjustments for covariates, no significant interaction was observed between liver function LCA group and liver resection range on the incidence of PHLF (*p* = 0.523). After adjusting for tumor number and blood loss, no significant interaction was found (*p* = 0.647). The association between liver function, LCA group, and PHLF remained largely consistent across different resection range subgroups, indicating result robustness.

## 4 Discussion

Risk stratification for PHLF and the planning of preoperative interventions present formidable challenges. Given the high mortality rate of PHLF, the preoperative identification of HCC patients at risk of liver dysfunction or failure is crucial [15, 16]. Previous studies of the prediction of liver failure have yielded multiple models and scores, such as the King's College Hospital criteria, Child–Turcotte–Pugh score (CTP), infection‐related organ failure, MELD, Chronic Liver Failure (CLIF)‐infection‐related organ failure, CLIF‐CACLFs, COSSH‐ACLH, and AARC‐ACLF scores [17, 18, 19, 20]. However, these prediction models have not been widely used due to the inclusion of subjective parameters, the restricted etiologic spectrum of included HCC cases, the limited verification of model universality, and inconsistent inclusion criteria. Clinical assessments of preoperative liver fibrosis, splenomegaly, and hypersplenism in patients with cirrhosis or chronic hepatitis have led to the development of imaging techniques for evaluating the liver and spleen, which may identify potential predictors of PHLF [21, 22].

This study innovatively applied LCA to precisely stratify 185 patients who underwent HCC resection. The incidence of PHLF was significantly higher in patients with poor liver function compared to those with good liver function (36.6% vs. 11.5%, respectively), and the extent of liver resection showed a threshold effect. These findings may inform the further exploration of the interaction between hepatic reserve and surgical trauma. Our multi‐dimensional evaluation system first confirmed that combinations of HBV status, degree of cirrhosis, spleen size, and PLT have greater predictive value for PHLF. Notably, the LCA model disclosed that patients with poor liver function exhibited characteristics similar to those with HBV‐related cirrhosis reported in previous studies; however, this study further quantified the risk level of PHLF in this subgroup, which is as high as 36.6%. This finding enhances the existing risk stratification system and provides a more reliable preoperative assessment tool. By comparing item‐response probabilities in Table S1, higher ICG R15 showed higher probabilities in the worse latent classes, indicating greater discriminative power for liver function stratification. This pattern aligns with an increased risk of PHLF, suggesting a stronger prognostic impact of ICG R15 on PHLF in a way. Furthermore, the range of resection remained an independent prognostic factor for PHLF in both crude and multivariable models. Additionally, to our knowledge, we report the first use of restricted cubic splines to determine the critical value for blood loss (150 mL), providing an objective standard for intraoperative blood loss management. The spline‐identified threshold of 150 mL reflects the point that achieves the most favorable sensitivity‐specificity balance. This cutoff also mirrors the low and relatively narrow distribution of intraoperative blood loss in our cohort under contemporary surgical techniques and effective anesthetic management. In clinical practice, the impact of PHLF can be modulated by several other factors, such as underlying liver disease, a limited functional future liver remnant (FLR) volume and others. Importantly, we consider 150 mL a cohort‐calibrated risk marker rather than an absolute clinical threshold and imply that when blood loss surpasses 150 mL, clinicians should be alert to the risk of PHLF and prioritize stabilization of vital signs and intravascular volume.

The clinical translational value of this study primarily comprises three aspects: First, the LCA classification system can facilitate the preoperative identification of high‐risk patients, particularly those with poor liver function (especially those with hepatitis B and cirrhosis). Surgeons should consider reducing the resection area or offering non‐surgical treatment options to these high‐risk patients. Second, the determination of a blood loss threshold provides a basis for formulating individualized surgical strategies. More importantly, our statistical model indicates that the impact of liver function grading on the resection range on PHLF is independent (interaction *p* > 0.05), suggesting that even after a small resection, patients with poor liver function still require enhanced postoperative monitoring. From a health economics perspective, precise risk stratification is expected to reduce intensive care unit (ICU) stays and rescue costs, which is especially crucial in resource‐limited settings. In the future bedside use, we can scale model coefficients to integer points to construct a bedside scoring system and a nomogram that map patients to the three LCA classes without running LCA software. We can further provide user‐friendly implementations, a nomogram and/or a web‐based calculator, thereby translating the LCA findings into a simple, clinician‐facing classification and risk tool for direct clinical use.

Despite significant progress, this study still has several limitations that must be addressed. The retrospective design may have introduced selection bias, particularly because the sample size (*n* = 185) meets statistical requirements but is insufficient for complex models such as potential category analysis, which could affect the stability of subgroup classification. However, the subgroup analysis performance was inadequate; consequently, future studies should expand the sample through multi‐center collaboration. Notably, the absence of an external validation cohort precludes the confirmation of the model's generalizability. To address these issues, we are preparing a prospective multi‐center study to conduct a more comprehensive validation. Long‐term follow‐up data will facilitate the assessment of the predictive value of this stratification system for perioperative complications and survival outcomes. In addition, we acknowledge that other intraoperative variables may also be risk factors for PHLF, such as operative time, duration of the Pringle maneuver, and transfusion requirements. However, these parameters were often ambiguously documented or incomplete, resulting in a high proportion of missing data. To avoid extensive case‐wise exclusion and the risk of selection bias, we did not include these variables in the primary analyses of the present study. These methodological limitations suggest that current conclusions are more suitable for generating hypotheses rather than directly guiding clinical practice.

This study demonstrates that the three‐dimensional hierarchical system of liver function based on LCA can effectively differentiate PHLF risk without interacting with the extent of liver resection. Although the incidence of PHLF was significantly higher in the poor liver function group, the 11.5% risk of PHLF among patients with good liver function group is notable, and suggests that surgical decisions should encompass multiple factors comprehensively. Future research should focus on three key areas: developing a dynamic prediction model incorporating imaging features; exploring critical blood loss in the context of big data; and developing preoperative liver function optimization strategies for high‐risk patients. These findings provide a new risk stratification framework for the design of precision surgery to treat patients with HCC.

## Author Contributions

**Ling Liu:** methodology, conceptualization, data curation, investigation, funding acquisition, writing – original draft, visualization. **Jintao Zheng:** data curation, investigation, software, writing – original draft. **Ye Wang:** project administration. **Chenao Yang:** supervision. **Jiachen Zhang:** supervision. **Changku Jia:** resources, funding acquisition, writing – review and editing, project administration.

## Funding

This work was supported by the Hangzhou Municipal Health Commission, Hangzhou Health Science and Technology Plan Project (grant number A20200526) and the Construction Fund of Key Medical Disciplines of Hangzhou (grant number 2025HZGF05).

## Ethics Statement

This study was approved by the Ethics Committee of the First Peoples Hospital of Hangzhou, affiliated with the Medical College of West Lake University (2025ZN162‐1). All participants provided written informed consent.

## Conflicts of Interest

The authors declare no conflicts of interest.

## Supporting information


**Figure S1:** Visualization results of the LCA three‐classification model.
**Figure S2:** Results of normality test for AFP, age, blood loss, BMI, and tumor size.
**Table S1:** Mean posterior probabilities, prevalence of latent calsses, and item‐response probabilites in models with two to four classes.

## Data Availability

Data related to patients that support the findings of this study are not publicly available due to privacy reasons but are available from the corresponding author upon reasonable request.
