# Supplementary material for: Association Between Liver Function Grade and Post‐Hepatectomy Liver Failure in Patients With Hepatocellular Carcinoma: A Latent Class Analysis
Source: Ann Gastroenterol Surg. 2025 Nov 25;10(3):819–26. doi: 10.1002/ags3.70138 (PMC13178270; doi:10.1002/ags3.70138)
Supplement: Supplementary file 1 — Figure S1: Visualization results of the LCA three‐classification model. Figure S2: Results of normality test for AFP, age, blood loss, BMI, and tumor size. Table S1: Mean posterior probabilities, prevalence of latent calsses, and item‐response probabilites in models with two to four classes. [file AGS3-10-819-s001.docx]

**Supporting Information**

**Title: Association between liver function grade and post-hepatectomy liver failure in patients with hepatocellular carcinoma: A latent class analysis**

*Ling Liu1,2#, Jintao Zheng1#, Ye Wang1, Chenao Yang3, Jiachen Zhang1, Changku Jia1,4**

** Corresponding author: Changku Jia*

*Department of Hepatobiliary and Pancreatic Surgery, The Fourth School of Clinical Medicine, Zhejiang Chinese Medical University; Email address: jiachk@126.com; Tel: 86-18248479988*

*
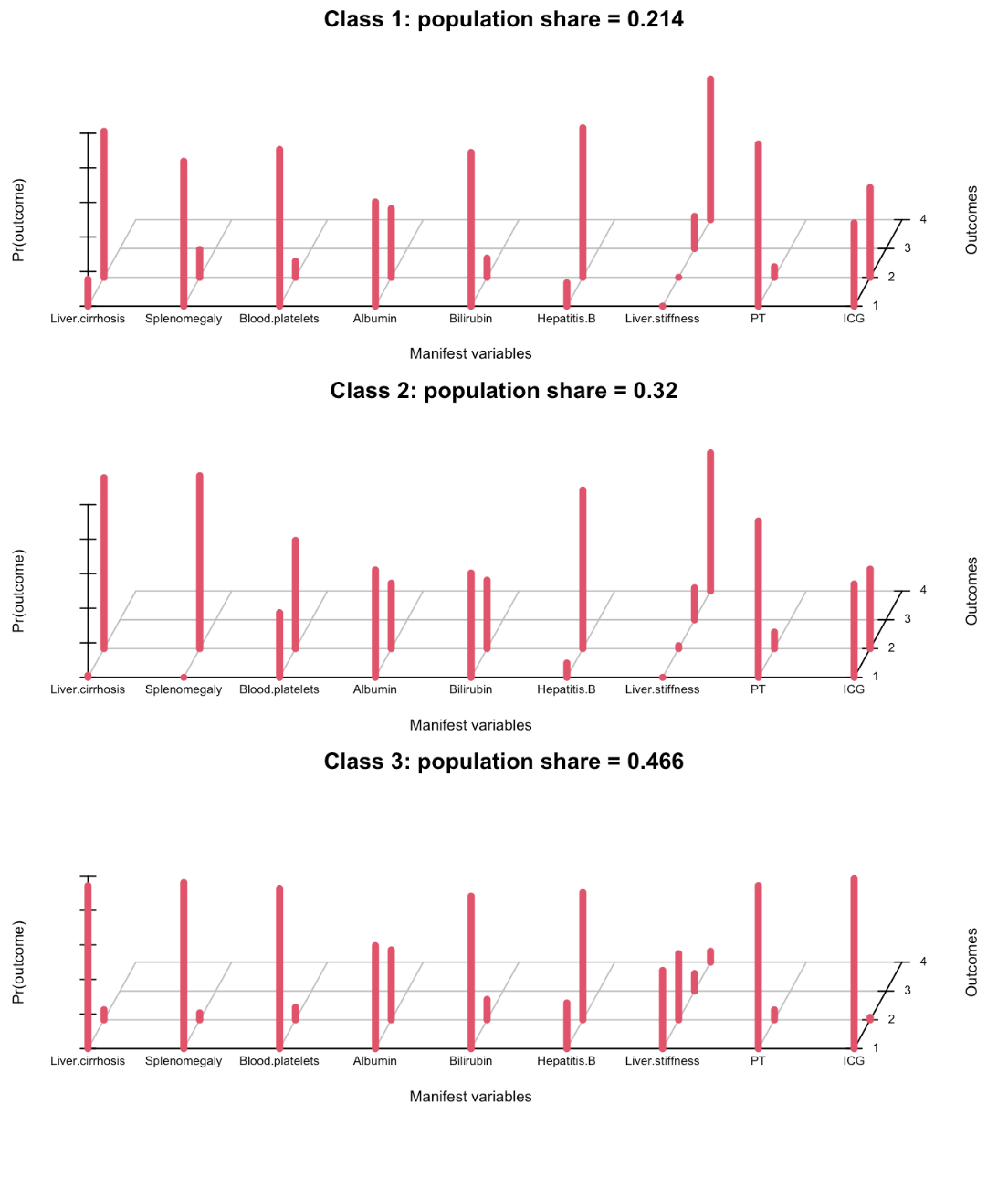
*

**Fig. S1** Visualization results of the LCA three-classification model

*
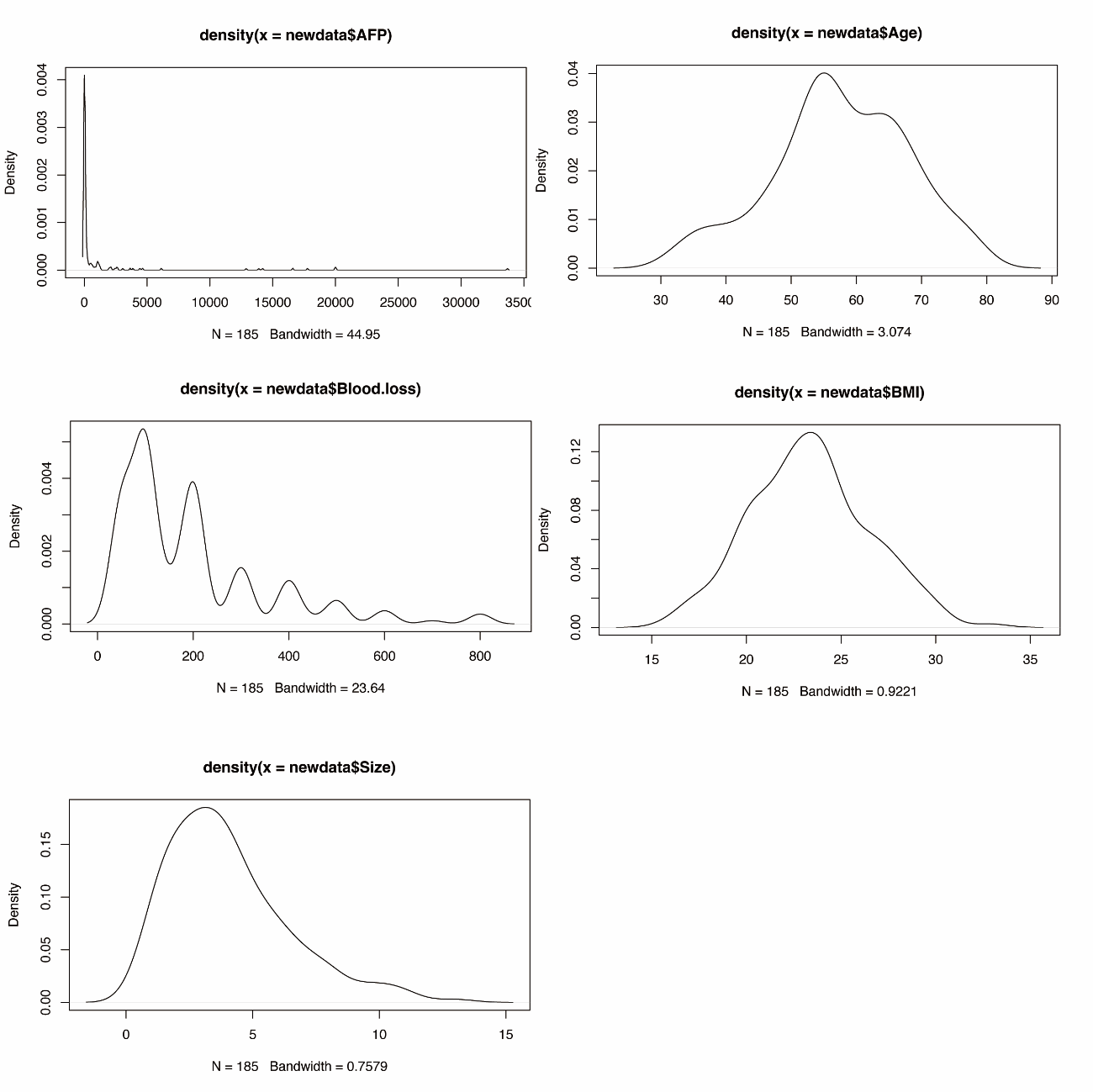
*

**Fig. S2** Results of normality test for AFP, age, blood loss, BMI, and tumor size

**Table S1. Mean posterior probabilities, prevalence of latent calsses, and item-response probabilites in models with two to four classes**

| **Item** | **Latent class1** | **Latent class2** | **Latent class3** | **Latent class4** |
| --- | --- | --- | --- | --- |
|  | Two-latent-class solution |  |  |  |
| MeanPP | 0.9683533 | 0.9919440 | NA | NA |
| Prevalence | 0.5243243 | 0.4756757 | NA | NA |
| Liver cirrhosis1 | 0.03871259 | 0.93332802 | NA | NA |
| Liver cirrhosis2 | 0.96128741 | 0.06667198 | NA | NA |
| Splenomegaly1 | 0.3057913 | 0.9634725 | NA | NA |
| Splenomegaly2 | 0.03994778 | 0.03652747 | NA | NA |
| Blood platelets1 | 0.5684340 | 0.9318627 | NA | NA |
| Blood platelets2 | 0.43156599 | 0.06813731 | NA | NA |
| Albumin1 | 0.6186805 | 0.5915018 | NA | NA |
| Albumin2 | 0.3813195 | 0.4084982 | NA | NA |
| Bilirubin1 | 0.7061625 | 0.8872137 | NA | NA |
| Bilirubin2 | 0.2938375 | 0.1127863 | NA | NA |
| Hepatitis B1 | 0.09894845 | 0.26156914 | NA | NA |
| Hepatitis B2 | 0.9010515 | 0.7384309 | NA | NA |
| Liver stiffness1 | 5.196106e-53 | 4.316031e-01 | NA | NA |
| Liver stiffness2 | 0.01248029 | 0.36319813 | NA | NA |
| Liver stiffness3 | 0.180265 | 0.110002 | NA | NA |
| Liver stiffness4 | 0.80725472 | 0.09519684 | NA | NA |
| PT1 | 0.9154081 | 0.9447295 | NA | NA |
| PT2 | 0.08459193 | 0.05527051 | NA | NA |
| ICG1 | 0.5086955 | 0.9722962 | NA | NA |
| ICG2 | 0.4913045 | 0.0277038 | NA | NA |
|  | Three-latent-class solution |  |  |  |
| MeanPP | 0.9616986 | 0.9077054 | 0.9726056 | NA |
| Prevalence | 0.3567568 | 0.4216216 | 0.2216216 | NA |
| Liver cirrhosis1 | 0.15530326 | 0.01104817 | 0.94208935 | NA |
| Liver cirrhosis2 | 0.84469674 | 0.98895183 | 0.05791065 | NA |
| Splenomegaly1 | 8.383895e-01 | 9.096378e-28 | 9.600514e-01 | NA |
| Splenomegaly2 | 0.1616105 | 1.0000000 | 0.0399486 | NA |
| Blood platelets1 | 0.9065680 | 0.3743457 | 0.9270264 | NA |
| Blood platelets2 | 0.09343204 | 0.62565433 | 0.07297361 | NA |
| Albumin1 | 0.6026586 | 0.6216881 | 0.5954948 | NA |
| Albumin2 | 0.3973414 | 0.3783119 | 0.4045052 | NA |
| Bilirubin1 | 0.8888319 | 0.6040338 | 0.8819896 | NA |
| Bilirubin2 | 0.1111681 | 0.3959662 | 0.1180104 | NA |
| Hepatitis B1 | 0.13503946 | 0.08253246 | 0.26411732 | NA |
| Hepatitis B2 | 0.8649605 | 0.9174675 | 0.7358827 | NA |
| Liver stiffness1 | 8.329490e-236 | 0.000000e+00 | 4.525707e-01 | NA |
| Liver stiffness2 | 7.869754e-55 | 1.654529e-02 | 3.831932e-01 | NA |
| Liver stiffness3 | 0.1861075 | 0.1843393 | 0.1011018 | NA |
| Liver stiffness4 | 0.81389251 | 0.79911538 | 0.06313426 | NA |
| PT1 | 0.9384685 | 0.9049084 | 0.9427415 | NA |
| PT2 | 0.06153152 | 0.09509158 | 0.05725848 | NA |
| ICG1 | 0.4809974 | 0.5405187 | 0.9857312 | NA |
| ICG2 | 0.5190026 | 0.4594813 | 0.0142688 | NA |
|  | Four-latent-class solution |  |  |  |
| MeanPP | 0.9233802 | 0.8832533 | 0.9713682 | 0.9224405 |
| Prevalence | 0.25405405 | 0.21081081 | 0.47027027 | 0.06486486 |
| Liver cirrhosis1 | 0.00000000 | 0.15081344 | 0.94457154 | 0.08836162 |
| Liver cirrhosis2 | 1.00000000 | 0.84918656 | 0.05542846 | 0.91163838 |
| Splenomegaly1 | 8.544398e-02 | 7.133875e-01 | 9.639752e-01 | 7.554472e-88 |
| Splenomegaly2 | 0.91455602 | 0.28661251 | 0.03602481 | 1.00000000 |
| Blood platelets1 | 0.2765137 | 1.0000000 | 0.9294372 | 0.3705935 |
| Blood platelets2 | 7.234863e-01 | 4.940656e-324 | 7.056278e-02 | 6.294065e-01 |
| Albumin1 | 0.5704155 | 0.6333713 | 0.5931254 | 0.7441643 |
| Albumin2 | 0.4295845 | 0.3666287 | 0.4068746 | 0.2558357 |
| Bilirubin1 | 0.5689366 | 0.8814799 | 0.8819981 | 0.7522247 |
| Bilirubin2 | 0.4310634 | 0.1185201 | 0.1180019 | 0.2477753 |
| Hepatitis B1 | 6.957264e-02 | 1.726119e-01 | 2.640536e-01 | 2.063175e-09 |
| Hepatitis B2 | 0.9304274 | 0.8273881 | 0.7359464 | 1.0000000 |
| Liver stiffness1 | 0.000000 | 0.000000 | 0.456062 | 0.000000 |
| Liver stiffness2 | 0.000000e+00 | 7.827816e-37 | 3.860194e-01 | 8.940673e-02 |
| Liver stiffness3 | 0.00000000 | 0.21090870 | 0.09570167 | 0.91059327 |
| Liver stiffness4 | 1.000000e+00 | 7.890913e-01 | 6.221695e-02 | 5.737021e-12 |
| PT1 | 0.8887573 | 0.9310574 | 0.9425062 | 1.0000000 |
| PT2 | 0.11124268 | 0.06894256 | 0.05749381 | 0.00000000 |
| ICG1 | 0.3718165 | 0.5599424 | 0.9853678 | 1.0000000 |
| ICG2 | 0.62818350 | 0.44005760 | 0.01463217 | 0.00000000 |
